# Microorganisms and clinical outcomes of early- and late-onset ventilator-associated pneumonia at Srinagarind Hospital, a tertiary center in Northeastern Thailand

**DOI:** 10.1186/s12890-021-01415-8

**Published:** 2021-01-30

**Authors:** Pavarit Arayasukawat, Apichart So-ngern, Wipa Reechaipichitkul, Worawat Chumpangern, Itthiphat Arunsurat, Pailin Ratanawatkul, Wanna Chuennok

**Affiliations:** 1 Department of Medicine, Srinagarind Hospital, Faculty of Medicine, Khon Kaen University, Khon Kaen, Thailand; 2 Division of Sleep Medicine, Srinagarind Hospital, Faculty of Medicine, Khon Kaen University, Khon Kaen, Thailand; 3 Division of Pulmonary and Critical Care Medicine, Srinagarind Hospital, Faculty of Medicine, Khon Kaen University, Khon Kaen, Thailand; 4 Infectious Control Unit, Srinagarind Hospital, Faculty of Medicine, Khon Kaen University, Khon Kaen, Thailand

**Keywords:** Early-onset VAP, Late-onset VAP, Microbiology, Outcome, Mortality

## Abstract

**Background:**

Ventilator-associated pneumonia (VAP) is a common nocosomial infection in intensive care unit (ICU). Local microbiological surveillance of pathogens and resistance patterns for early-onset VAP (EOVAP) and late-onset VAP (LOVAP) will help to choose appropriate empiric antibiotics.

**Objective:**

To compare the multi-drug resistant (MDR) pathogens, treatment outcomes, and factors associated with hospital mortality of VAP.

**Method:**

A cross-sectional study between 1 January 2015 and 31 December 2017 at Srinagarind hospital, Khon Kaen University was conducted. The demographic data, causative pathogens, hospital length of stay (LOS), ICU LOS, mechanical ventilator (MV) days, and hospital mortality were retrospectively reviewed.

**Results:**

One hundred and ninety patients were enrolled; 42 patients (22%) were EOVAP and 148 patients (78%) were LOVAP. *Acinetobacter baumannii* was the most common pathogen in both groups (50% EOVAP vs 52.7% LOVAP). MDR pathogens were significant greater in LOVAP (81.8%) than EOVAP (61.9%) (*p* = 0.007). The EOVAP had a significantly better ICU LOS [median (interquartile range, IQR) 20.0 (11.0, 30.0) vs. 26.5 (17.0, 43.0) days], hospital LOS [median (IQR) 26.5 (15.0, 44.0) vs. 35.5 (24.0, 56.0) days] shorter MV days [median (IQR) 14.0 (10.0, 29.0) *vs*. 23.0 (14.0, 35.5) days] and lower hospital mortality (16.7% vs 35.1%) than LOVAP (*p* < 0.05). The factor associated with hospital mortality was having simplified acute physiology (SAP) II score ≥ 40 with an adjusted odds ratio (aOR) of 2.22 [95% confidence interval (CI), 1.08–4.54, *p* = 0.02].

**Conclusion:**

LOVAP had significantly higher MDR pathogens, MV days, ICU LOS, hospital LOS and hospital mortality than EOVAP. A broad-spectrum antibiotic to cover MDR pathogens should be considered in LOVAP. The factor associated with hospital mortality of VAP was a SAPII score ≥ 40.

## Background

Pneumonia is the most common hospital-acquired infection with a prevalence of approximately 22% [1, 2]. Ventilator-associated pneumonia (VAP) is pneumonia developing after 48–72 h of endotracheal intubation [3–5]. VAP is the most common nosocomial infection, developed in about 5–40% of mechanically ventilated patients [5–7]. Data from the International Nosocomial Infection Control Consortium (INICC) collected summary data from 50 countries including Southeast Asia during 2010–2015 indicated the VAP rate was 13.1 per 1000 mechanical ventilator (MV) days in the medical and surgical intensive care unit (ICU) [8]. Similar results of Reechaipichitkul et al. who determined that VAP rates in Srinagarind Hospital, Khon Kaen University, a tertiary-care hospital in northeastern Thailand were 13.6 and 12.6 per 1000 MV days in 2008 and 2009. This study also demonstrated that more than half of the costs of nocosomial treatment in 2008 and 2009 were the costs for hospital acquired pneumonia (HAP) and VAP, 16.8 and 17.5 million Baht [9]. Melsen et al. performed a meta-analysis and suggested that overall attributable mortality in mechanical ventilator patients from VAP was 13% [10].

VAP was categorized into early-onset VAP (EOVAP) and late-onset VAP (LOVAP) depending upon when it occurred on which days after hospitalization. The cutoff point of a range 4–7 days onset varied across the studies [11–16]. Recent guideline for HAP and VAP management from The Infectious Disease Society of America (IDSA)/American Thoracic Society (ATS) and the International ERS/ESICM/ESCMID/ALAT use the cutoff point of 5 days after hospitalization [2, 17, 18]. It is believed that in EOVAP, the causative pathogens are not drug-resistant bacteria such as *Streptococcus pneumoniae*, *Haemophilus influenzae,* antibiotic-sensitive enteric gram-negative bacilli or methicillin-sensitive *Staphylococcus aureus* (MSSA). There is a greater risk that the causative pathogens in LOVAP are multi-drug resistant (MDR) such as *Acinetobacter baumannii*, *Pseudomonas aeruginosa*, methicillin-resistant *S. aureus* (MRSA), extended-spectrum beta-lactamase (ESBL)-producing bacteria and other gram-negative bacilli [5, 17, 19, 20]. The prevalence of MDR pathogens between EOVAP and LOVAP in several studies remained a controversy. Several studies demonstrated that EOVAP had a significantly lower prevalence of MDR pathogens [21–23]. Subsequent studies, however, did not show a significant difference in MDR pathogens between EOVAP and LOVAP groups [11, 12, 14, 24].

Therefore, this study was conducted and aimed to compare the pathogens, clinical characteristics, treatment outcomes between EOVAP and LOVAP groups, and factors associated with hospital mortality.

## Methods

A cross-sectional study between 1 January 2015 and 31 December 2017 was conducted at Srinagarind Hospital, Faculty of Medicine, Khon Kaen University, a 1466-bed tertiary care center in Northeast Thailand. In our hospital, patients who underwent mechanical ventilator received VAP bundle care as following: (1) endotracheal tube suctioning every 2 h, (2) sedation keeping Richmond Agitation Sedation Scale (RASS) of 0 to − 1, (3) oral decontamination with 0.2% chlorhexidine, (4) aspiration precaution with head elevation of 30°–45° and (5) hand hygiene either with 4% chlorhexidine soap and water or with alcohol-based hand rub. In a case of VAP development; infection control ward nurses (ICWNs) reported all patients’ data to infectious control (IC) unit system. We retrieved all VAP patient data from these recordings. The study was approved by the Human Research Ethics Committee, Khon Kaen University (approval number HE611281).

### Study subjects

VAP was diagnosed by the following criteria: (1) a pulmonary infection occurring 48 h after mechanical ventilation (2) new pulmonary infiltration on chest radiograph (3) at least two of the three following characteristics: temperatures > 38.3 °C or < 36.5 °C, purulent tracheal secretions, and leukocytosis (white blood cell > 12,000 cells/mm^3^) or leukopenia (white blood cell < 4000 cells/mm^3^) [4, 25]. The exclusion criteria were as following: (1) patients who had previous abnormal chest imaging including pulmonary edema, adult respiratory distress syndrome, pulmonary embolism, alveolar hemorrhage, pulmonary tuberculosis, and recent pneumonia. (2) Immunocompromised patients who received any immunosuppressive agents, chemotherapy, or prednisolone equivalence ≥ 15 mg/day.

### Data collection

The medical records of demographic data, hospital department, laboratory results, chest radiological findings, microbiological profiles, tracheostomy tube placement, hospital length of stay (LOS), intensive care unit (ICU) LOS, mechanical ventilator (MV) days and hospital mortality were reviewed.

### Definition and outcome

EOVAP was VAP developed before 5 calendar days of hospitalization while LOVAP was VAP occurred at least 5 calendar days of hospitalization. MDR bacteria were defined as organisms that resisted at least 3 classes of antibiotics [26]. MDR pathogens included ESBL-producing bacteria, carbapenem-resistant Enterobacteriaceae (CRE), MRSA, and other MDR bacteria that were reported from the microbiological laboratory. The causative pathogens were defined as one or more of the following: (1) an isolated organism from hemoculture (2) an isolated organism from pleural effusion (3) an isolated numerous growth organism on a semiquantitative method or isolated organism on the quantitative method i.e. endotracheal aspirate > 10^5^ colony-forming unit (CFU)/ml, bronchoalveolar lavage > 10^4^ CFU/ml or protected specimen brush ≥ 10^3^ CFU/ml. Hospital mortality was death occurring during the same admission of VAP diagnosis.

Empiric antibiotic therapy was prescribed according to local antibiogram and local data [9]. The commonly used empiric antibiotics for VAP were carbapenems, colistin and piperacillin/tazobactam. The causative pathogens and drug susceptibility tests were reported approximately 72 h after treatment. The proper antibiotic use was defined as the causative pathogens were susceptible to the initial empiric antibiotics.

The primary outcome was to compare the MDR pathogens between EOVAP and LOVAP. The secondary outcomes were to compare causative pathogens, hospital length of stay (LOS), ICU LOS, MV days, and hospital mortality between EOVAP and LOVAP. Factors associated with hospital mortality of VAP were identified.

### Statistical analysis

The categorical data were shown as numbers and percentages. The normal distributed continuous data were presented as mean and standard deviation (SD) while the non-normal distributed data were presented as the median and interquartile range (IQR). A comparison of category data used the Chi-square test and Fisher’s exact test depending on data. The nonparametric data used the Mann–Whitney U test for comparison. The factors associated with hospital mortality in VAP subjects were evaluated by univariate logistic regression analysis. The stepwise backward multiple logistic regression analysis of factors with a *p*-value < 0.2 on univariate analysis or factors with previous reports of clinical significance were performed. Crude odds ratio (cOR) and adjusted odds ratio (aOR) with their 95% confidence intervals (CI) were demonstrated. A *p*-value of less than 0.05 was considered statistically significant. The statistical analysis was performed by Stata version 10.1 (StataCorp, College Station Texas, USA).

## Results

### Patients

During the study period, 190 patients were diagnosed with VAP. Forty-two patients were EOVAP and 148 patients were LOVAP. The mean (SD) age of these was 64.3 (16.2) years. Males were 127 patients (66.8%) and females were 63 patients (33.2%). One hundred and seven patients (56.3%) were admitted to the Medicine Department (96 patients (50.5%) in medical ICU ward and 11 patients (5.8%) in general medicine ward). Eighty-three patients (43.7%) were admitted to the Surgical Department (73 patients (38.4%) in surgical ICU ward and 10 patients (5.3%) in general surgery ward). One hundred and forty-eight patients (77.9%) had an underlying disease. The common underlying diseases were hypertension (41.6%), diabetes mellitus (27.4%), cardiovascular disease (26.8%). The mean (SD) of the simplified acute physiology (SAP) II score was 43.7 (13.3). Lobar pneumonia was the most common finding on chest radiography which was found in 145 patients (76.3%). Pleural effusion developed in 54 patients (28.4%). The demographic data of EOVAP and LOVAP patients were shown in Table 1. LOVAP patients had a higher mean age and more comorbidities than EOVAP patients while the chest radiographic findings were similar between groups.

**Table 1:** Demographic data of early-onset VAP (n = 42) and late-onset VAP (n = 148)

Characteristics	Early-onset VAP n (%)	Late-onset VAP n (%)
Mean age in years (SD)	58.5 (16.9)	65.9 (15.7)
Male	34 (81)	93 (62.8)
Ward		
Medical ICU	14 (33.3)	82 (55.4)
Surgical ICU	21 (50.0)	52 (35.1)
General medicine ward	3 (7.1)	8 (5.4)
General surgery ward	4 (9.5)	6 (4.1)
Underlying diseases	28 (66.7)	120 (81.1)
Hypertension	17 (40.5)	62 (41.9)
Diabetes mellitus	10 (23.8)	42 (28.4)
Cardiovascular disease	11 (26.2)	40 (27.0)
Renal failure	4 (9.5)	37(25.0)
Neurological disease	6 (14.3)	22 (14.9)
Dyslipidemia	4 (9.5)	17 (11.5)
Lung disease	6 (14.3)	13 (8.8)
Gastrointestinal disease	2 (4.8)	11(7.4)
Other	1 (2.4)	17 (11.5)
Hospitalized within 90 days	4 (9.5)	10 (6.8)
Antibiotic therapy in the prior month	22 (52.4)	101 (68.2)
Mean SAP II score (SD)	40.9 (14.1)	44.4 (12.9)
Chest radiographic finding		
Lobar pneumonia	34 (80.9)	111 (75.0)
Multilobar pneumonia	8 (19.0)	37 (25.0)
Pleural effusion	12 (28.6)	42 (28.4)

*VAP* ventilator-associated pneumonia, *SD* standard deviation, *ICU* intensive care unit, *IQR* interquartile range, *SAP II score* simplified acute physiology II score

### Primary outcome

The causative pathogens were mostly gram-negative bacteria (97.4%) while gram-positive bacteria were isolated 2.6%. The most common pathogens were *A. baumannii* (52.1%), *Klebsiella pneumoniae* (15.3%), *Stenotrophomonas maltophilia* (13.2%), *P. aeruginosa* (8.9%). The MDR pathogens were identified 77.4%; 3.7% of ESBL-producing bacteria, 5.3% of CRE, 1.6% of MRSA and 66.8% of other MDR gram-negative bacteria. The MDR bacteria were found 61.9% in the EOVAP and 81.8% in LOVAP. The LOVAP had significantly more MDR pathogens than EOVAP (*p* = 0.007). The data were shown in Table 2. The proper antibiotics were used to treat 130 patients (68.4%); 26 patients (61.9%) of EOVAP and 104 patients (70.3%) of LOVAP. The proportion of proper antibiotics was similar between groups (*p* = 0.30).

**Table 2:** Microorganisms identified in early-onset VAP (n = 42) and late-onset VAP (n = 148)

Microorganism	Early-onset VAP n (%)	Late-onset VAP n (%)	p-value
Gram-negative organism	40 (95.2)	145 (97.9)	0.31
* Acinetobacter baumannii*	21 (50.0)	78 (52.7)	0.76
MDR *Acinetobacter baumannii*	20 (47.6%)	73 (49.3)	0.84
* Klebsiella pneumoniae*	8 (19.0)	21 (14.2)	0.44
MDR *Klebsiella pneumoniae*	1 (2.4)	18 (12.2)	
ESBL-*Klebsiella pneumoniae*	0 (0.0)	5 (3.4)	
CRE *Klebsiella pneumoniae*	1 (2.4)	9 (6.1)	
* Pseudomonas aeruginosa*	3 (7.1)	14 (9.5)	0.64
MDR *Pseudomonas aeruginosa*	1 (2.4)	2 (1.4)	
* Stenotrophomonas maltophilia*	2 (4.8)	23 (15.5)	0.07
MDR *Stenotrophomonas maltophilia*	2 (4.8)	22 (14.9)	
* Enterobacter *spp.	2 (4.8)	2 (1.4)	0.17
MDR *Enterobacter *spp.	1 (2.4)	2 (1.4)	
ESBL-*Enterobacter *spp.	1 (2.4)	1 (2.4)	
Other gram-negative organisms	4 (1.7)	7 (4.73)	
Gram-positive organism	2 (4.8)	3 (2.0)	0.24
*Staphylococcus aureus*	1 (2.4)	2 (1.4)	0.31
MRSA	1 (2.4)	2 (1.4)	0.64
Other gram-positive organisms	1 (2.4)	1 (0.7)	0.33
Multidrug-resistant pathogens**	26 (61.9)	121 (81.8)	0.007*

*VAP* ventilator-associated pneumonia, *ESBL* extended-spectrum beta-lactamase, *CRE* carbapenem-resistant Enterobacteriaceae, *MRSA* methicillin-resistant *Staphylococcus aureus*

*p-value < 0.05

**Multidrug-resistant pathogens included ESBLs, CRE, MRSA, and other MDR organisms

### Secondary outcomes

The median (IQR) duration of MV day was 22.0 (12.0, 34.0) days. The median (IQR) duration of MV day was significantly longer in LOVAP [23.0 (14.0, 35.5) vs 14.0 (10.0, 29.0) days); *p* = 0.03). The median (IQR) ICU LOS was 25.0 (15.0, 42.0) days. The median (IQR) ICU LOS was significantly longer in LOVAP [26.5 (17.0, 43.0) vs 20.0 (11.0, 30.0) days; *p* = 0.02]. The median (IQR) hospital LOS was 34.0 (23.0, 53.0) days. The median (IQR) hospital LOS was significantly longer in LOVAP [35.5 (24.0, 56.0) vs 26.5 (15.0, 44.0) days; *p* = 0.01]. Tracheostomy was performed in 30.5% (38.1% of EOVAP and 28.4% of LOVAP). The overall hospital mortality during the study period was 31.1%. The hospital mortality was significantly greater in LOVAP (35.1% vs 16.7%; *p* = 0.02). The data were shown in Table 3.

**Table 3:** Outcomes of treatment in early-onset VAP (n = 42) and late-onset VAP (n = 148)

Outcomes	Early-onset VAP	Late-onset VAP	p-value
Median duration MV day (day, IQR)	14.0 (10.0, 29.0)	23.0 (14.0, 35.5)	0.03*
Median ICU LOS (day, IQR)	20.0 (11.0, 30.0)	26.5 (17.0, 43.0)	0.02*
Median hospital LOS (day, IQR)	26.5 (15.0, 44.0)	35.5 (24.0, 56.0)	0.01*
Performed tracheostomy (n. %)	16.0 (38.1)	42.0 (28.4)	0.22
Hospital mortality (n, %)	7.0 (16.7)	52.0 (35.1)	0.02*

*VAP* ventilator-associated pneumonia, *MV* mechanical ventilator, *ICU* intensive care unit, *IQR* interquartile range, *LOS* length of stay

*p-value < 0.05

### Factor associated hospital mortality

Univariate and multivariate analysis were performed to assess factors associated with hospital mortality. On univariate analysis, the patients who were of an age ≥ 60 years (cOR = 2.19; 95% CI 1.11–4.33; *p* = 0.02), were admitted in the medical ICU (cOR = 2.28; 95% CI 1.20–4.29; *p* = 0.01), having a SAPII score ≥ 40 ICU (cOR = 2.49; 95% CI 1.28–4.86; *p* = 0.007), receiving improper antibiotics (cOR = 2.27; 95% CI 1.10–4.68; *p* = 0.02), or were late-onset VAP (cOR = 2.71; 95% CI 1.12–6.52; *p* = 0.02) were statistically associated with hospital mortality of VAP patients. On stepwise backward multivariate analysis, having a SAPII score ≥ 40 was the statistically significant factor associated with hospital mortality (aOR = 2.22; 95% CI 1.08–4.54; *p* = 0.02). The data were shown in Table 4.

**Table 4:** Factors associated with hospital mortality in VAP patients

Factors	Crude OR (95% CI)	Adjusted OR (95% CI)	p-value
Age ≥ 60 years	2.19 (1.11–4.33)	–	0.02
Having underlying diseases	0.99 (0.47–2.08)	–	0.99
Patient at medical ICU	2.28 (1.20–4.29)	–	0.01
Having SAP II score ≥ 40	2.49 (1.28–4.86)	2.22 (1.08–4.54)	0.02*
Resistant gram-negative organisms	1.04 (0.51–2.13)	–	0.92
Receiving improper antibiotics	2.27 (1.10–4.68)	–	0.02
Late-onset VAP	2.71 (1.12–6.52)	–	0.02

*VAP* ventilator-associated pneumonia, *OR* odds ratio, *CI* confidence interval, *ICU* intensive care unit, *SAP II score* simplified acute physiology II score

*p-value for 95% CI of adjusted OR

## Discussion

The study revealed that the most common pathogens were gram-negative bacteria. *A. baumannii*, *K. pneumoniae*, *P. aeruginosa* were common pathogens in both groups while *S. maltophilia* was increased in LOVAP. The pathogens from this study did not differ between EOVAP and LOVAP. The results of this study were similar to other tertiary centers in Thailand [27, 28]. Of these, *A. baumannii*, *K. pneumoniae*, *P. aeruginosa* were the common pathogens of VAP. These studies, however, did not address the causative pathogens into EOVAP and LOVAP. Three studies from different tertiary-care centers of India had results similar to our study [14, 15, 29]. *A. baumannii*, *K. pneumonia* and *P. aeruginosa* were common pathogens in both EOVAP and LOVAP. The pathogens of EOVAP from this study differed from pathogens mentioned in the recent guideline [17]. The results supported that empiric antibiotics should be guided by a local distribution of pathogens that are recommended by the Management of Adults with Hospital-acquired and Ventilator-associated Pneumonia in 2016 by IDSA/ATS guideline [2]. Papazian et al. suggested that microbiological confirmation is strongly recommended when considering a diagnosis of VAP and pathogens may vary depending on many factors including the duration of MV, hospital LOS, ICU LOS, previous antibiotics exposure, the occurrence of epidemic phenomena in a given ICU and local distribution of organisms [5].

Gram-positive bacteria were identified in only 2.6% and most of them were MRSA. The prevalence of drug-resistance gram-positive bacteria in this study was markedly lower as compared to the study of the pathogens of VAP in Thailand by Chittawatanarat et al., Inchai et al. and Werarak et al. [27, 28, 30]. Reechaipichitkul et al. conducted a study of the causative organisms of VAP in the same center during 2008–2009. The study indicated MRSA was responsible for 6–7% of the total causative pathogens [9]. The majority of *S. aureus* colonization in the respiratory tract is in the nares and throat. Chlorhexidine is a topical antiseptic, which is most active against gram-positive bacteria [31]. Our center has applied selective oral decontamination (SOD) with chlorhexidine since 2011. This might have reduced the incidence of VAP due to MRSA.

Inappropriate and delayed empiric antibiotics are associated with higher mortality in VAP patients [32–34]. In our center, the empiric antibiotic was based on the previous local study in 2008 [9]. The study demonstrated that gram-negative bacteria were the majority of VAP causative pathogen. The three most common pathogens were *A. baumannii*, *P. aeruginosa* and *K. pneumoniae*. *A. Baumannii* mostly resisted carbapenems, cefoperazone/sulbactam, piperacillin/tazobactam but were still susceptible to colistin. *P. aeruginosa* resisted mostly to carbapenems but were still susceptible to ceftazidime, piperacillin/tazobactam and levofloxacin. Forty-seven percent of *K. pneumoniae* was ESBL producer. The carbapenems had activity against ESBL producing *K. pneumoniae* [9]. The purpose of differentiation of VAP into EOVAP and LOVAP was to guide empiric antibiotic treatment to cover MDR bacteria. The study found that LOVAP had a significantly higher proportion of MDR pathogens than EOVAP (*p* = 0.007). The results endorsed the Management of Adults with Hospital-acquired and Ventilator-associated Pneumonia in 2016 by IDSA/ATS suggested that VAP developed after 5 days of hospitalization had a greater risk of MDR pathogen presence than VAP developed earlier [2]. Therefore empiric broad-spectrum antibiotics against MDR pathogens were recommended for LOVAP.

Furthermore, this current study demonstrated that LOVAP had significantly longer MV days, ICU LOS, and hospital LOS than EOVAP. The hospital mortality was significantly greater in LOVAP (35.1% vs 16.7%, *p* = 0.02). These worse outcomes of LOVAP were also observed by Khan et al. [24]. The implementation of VAP prevention might reduce the cost of hospitalization and unnecessary mortality, especially in LOVAP [35].

A meta-analysis from Melsen et al. suggested that overall attributable mortality from VAP was 13% and the higher mortality were found in surgical patients, acute physiology and chronic health evaluation (APACHE) score of 20–29 and SAPS II score of 35–58 [10]. Bekaert et al.revealed the SAPS II score of 28–40 was significantly greatest associated with ICU death per additional day since the onset VAP [36]. Similar to our study, on stepwise backward multivariate analysis, a SAPII score ≥ 40 was significantly associated with hospital mortality of VAP patients.

The strengths of this study were that the recorded data were complete because VAP was under regular surveillance of our institute by ICWNs and confirmed by the IC unit.

This study had some limitations. First, the sample size is small, especially in EOVAP. This affected the statistical power. Second, this was a retrospective study, some data might be difficult to determine such as previous antibiotic exposure within 90 days, prior hospitalization preceding 90 days. These factors are associated with MDR pathogen infections [2, 37]. Third, the results of this study were unable to be applied to VAP in immunocompromised patients. Fourth, this study was from a single tertiary center, which had some limitations for the application in general hospitals. Pathogens and resistance patterns could vary between hospitals, regions and countries [2]. The local pathogens and pattern resistance of each hospital were the crucial factors for the selection of initial empiric antibiotics.

## Conclusion

In conclusion, LOVAP was significantly higher MDR pathogens, MV days, ICU LOS, hospital LOS and hospital mortality than EOVAP. A broad-spectrum antibiotic to cover MDR pathogens should be considered in LOVAP. The factor associated with hospital mortality of VAP was a SAPII score ≥ 40.

## Data Availability

The datasets used and/or analyzed in this study are available from the corresponding author on reasonable request.

## Abbreviations

VAP: Ventilator-associated pneumonia
ICU: Intensive care unit
EOVAP: Early-onset ventilator-associated pneumonia
LOVAP: Late-onset ventilator-associated pneumonia
LOS: Length of stay
MV: Mechanical ventilator
MDR: Multi-drug resistant
SAP II score: Simplified acute physiology II score
INICC: International Nocosomial Infection Control Consortium
IDSA: Infectious Disease Society of America
ATS: American Thoracic Society
ERS: European Respiratory Society
ESICM: European Society of Intensive Care Medicine
ESCMID: European Society of Clinical Microbiology and Infectious Diseases
ALAT: Latin American Thoracic Association
IC: Infectious control
ICWNs: Infection control ward nurses
SOD: Selective oral decontamination
MSSA: Methicillin-sensitive *Staphylococcus aureus*
MRSA: Methicillin-resistant *Staphylococcus aureus*
ESBL: Extended-spectrum beta-lactamase
CRE: Carbapenem-resistant Enterobacteriaceae
CFU: Colony-forming unit
ml: Millilitre
mg: Milligram
SD: Standard deviation
IQR: Interquartile range
aOR: Crude odds ratio
cOR: Adjusted odds ratio
CI: Confidence interval
